# AI in Hand Surgery: Assessing Large Language Models in the Classification and Management of Hand Injuries

**DOI:** 10.3390/jcm13102832

**Published:** 2024-05-11

**Authors:** Sophia M. Pressman, Sahar Borna, Cesar A. Gomez-Cabello, Syed Ali Haider, Antonio Jorge Forte

**Affiliations:** 1 Division of Plastic Surgery, Mayo Clinic, Jacksonville, FL 32224, USA; 2 Center for Digital Health, Mayo Clinic, Rochester, MN 55905, USA

**Keywords:** artificial intelligence (AI), ChatGPT, Gemini, deep learning, machine learning, hand surgery, hand trauma, management

## Abstract

**Background**: OpenAI’s ChatGPT (San Francisco, CA, USA) and Google’s Gemini (Mountain View, CA, USA) are two large language models that show promise in improving and expediting medical decision making in hand surgery. Evaluating the applications of these models within the field of hand surgery is warranted. This study aims to evaluate ChatGPT-4 and Gemini in classifying hand injuries and recommending treatment. **Methods**: Gemini and ChatGPT were given 68 fictionalized clinical vignettes of hand injuries twice. The models were asked to use a specific classification system and recommend surgical or nonsurgical treatment. Classifications were scored based on correctness. Results were analyzed using descriptive statistics, a paired two-tailed *t*-test, and sensitivity testing. **Results**: Gemini, correctly classifying 70.6% hand injuries, demonstrated superior classification ability over ChatGPT (mean score 1.46 vs. 0.87, *p*-value < 0.001). For management, ChatGPT demonstrated higher sensitivity in recommending surgical intervention compared to Gemini (98.0% vs. 88.8%), but lower specificity (68.4% vs. 94.7%). When compared to ChatGPT, Gemini demonstrated greater response replicability. **Conclusions**: Large language models like ChatGPT and Gemini show promise in assisting medical decision making, particularly in hand surgery, with Gemini generally outperforming ChatGPT. These findings emphasize the importance of considering the strengths and limitations of different models when integrating them into clinical practice.

## 1. Introduction

The integration of artificial intelligence (AI) into daily medical practice is an evitable reality [1,2]. With an expanding repertoire of applications and tools, AI is infiltrating the healthcare landscape, seeping into every specialty and healthcare domain [3,4]. This progression is marked by the emergence of large language models (LLMs). Built upon neural network architectures [5], LLMs are AI systems which are designed to employ deep learning and natural language processing [6]. These models undergo extensive training on vast datasets until they are able to comprehend and generate human-like text with significant predictability [6], even without employing retrieval-augmented generation (RAG) approaches. Two publicly available LLMs that have garnered recent interest are Google’s Gemini [7] (built upon its predecessor Bard) and OpenAI’s ChatGPT [8]. Although not designed specifically for medical purposes, these LLMs have demonstrated potential in medical decision making [9]. For example, ChatGPT achieved passing scores for both the United States Medical Licensing Examination [10] and American Society for Surgery of the Hand self-assessment exam [11], demonstrating a basic level of competence in medical reasoning. With the potential to enhance diagnostic accuracy, informed decision making, and patient outcomes, LLMs like ChatGPT and Gemini show promise in augmenting healthcare delivery [9,12].

The field of hand surgery stands to benefit greatly from the support of AI tools like LLMs [1,13]. Achieving favorable outcomes after hand trauma is paramount, given the critical role that hands play in necessary tasks for daily functioning and independence [14,15,16,17]. Effective management relies heavily on correctly identifying the nature and severity of injuries, as this information guides decisions regarding surgical intervention, rehabilitation, and ongoing care [18,19,20,21]. Inaccurate diagnosis and classification can lead to delays in treatment, inappropriate interventions, and potentially compromised functional recovery for patients [22,23]. With their advanced algorithms, LLMs can analyze complex patterns [6] and thus have the potential to provide rapid and precise injury classifications, enhancing and expediting the management process.

While the integration of advanced LLMs into healthcare systems is on the horizon, access may be limited to those within those institutions. Healthcare professionals in underfunded facilities, medical students, and patients are expected to continue using publicly available LLMs like ChatGPT and Gemini for medical queries. The continuous assessment of the value and utility of these LLMs is essential. Early investigations into LLM applications in hand surgery have shown promising results. Leypold et al. [24] found that ChatGPT adeptly managed complex hand and arm surgery scenarios, showcasing the potential of LLMs like ChatGPT to improve patient care and surgical outcomes. Crook et al. [25] reported ChatGPT’s proficiency in addressing common patient inquiries regarding common hand surgeries, noting generally high-quality responses. In a similar study, Seth et al. [26] found that ChatGPT was suitable for nonmedical individuals, but struggled with accurate and complete references. Additionally, Al Rawi et al. [27] observed that, while ChatGPT’s responses were correct and useful in most cases, only 57% were deemed complete by hand surgeon reviewers.

Although these studies show promise for ChatGPT, there is limited research exploring Gemini’s potential in hand surgery and few studies comparing LLMs. Furthermore, additional research to explore the full extent of LLM capabilities in the context of hand injuries and hand surgery, particularly in specialized tasks like injury classification, is warranted. The objective of this study is to assess the ability of ChatGPT and Gemini to accurately classify hand injuries via the use of 12 specific classification systems. Furthermore, this study aims to determine the ability of these models to accurately recommend surgical or nonsurgical management for these hand injuries. In doing so, we seek to evaluate the capabilities of publicly available LLMs without the use of RAG approaches. Through this investigation, this study endeavors to advance the ongoing discourse surrounding the applications and limitations of LLMs in hand surgery.

## 2. Materials and Methods

### 2.1. Study Design

Sixty-eight unique prompts were developed to test each LLM’s ability to classify hand injuries. Prompts cover 12 different classification systems [20,21,28,29,30,31,32,33,34,35,36,37,38,39] covering various hand injuries. The inclusion criteria prioritized classification systems with well-established significance, ensuring relevance and familiarity among healthcare providers specializing in hand surgery. To focus on clinically relevant systems, classification systems lacking direct treatment correlations were excluded.

**Figure 1:**
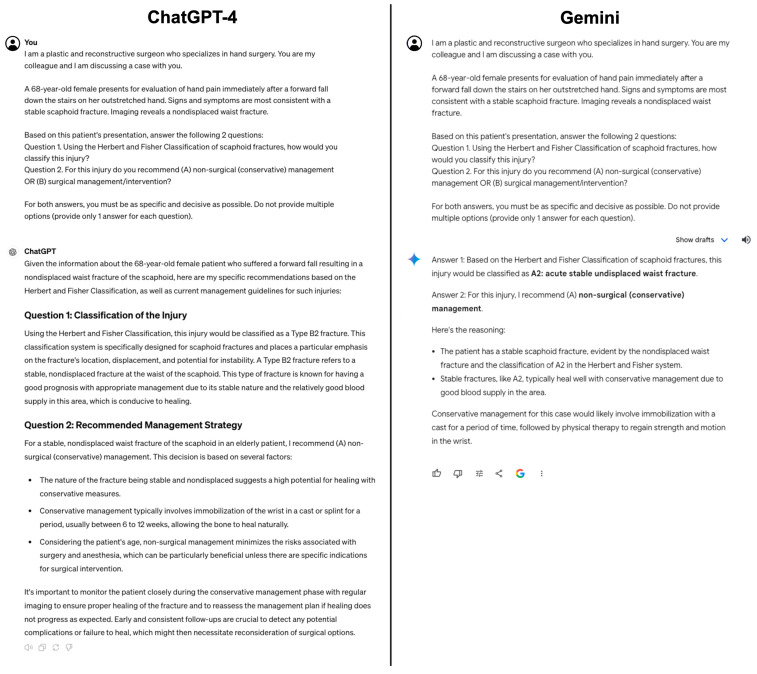
An example of a prompt given to ChatGPT-4 (**left**) and Gemini (**right**) with the corresponding responses below. This prompt asked the models to classify a scaphoid fracture using Herbert and Fisher’s classification system.

**Figure 2:**
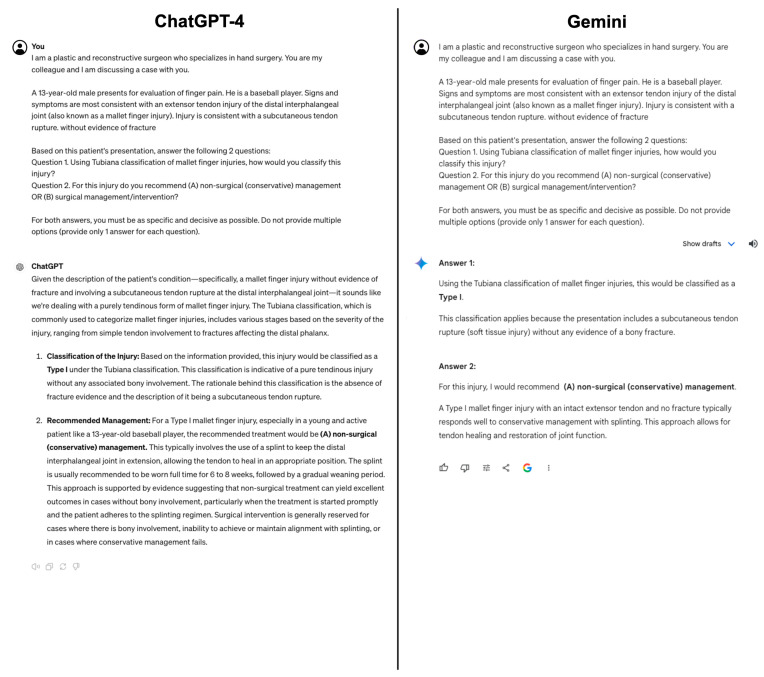
An example of a prompt given to ChatGPT-4 (**left**) and Gemini (**right**) with the corresponding responses. This prompt asked the models to classify a mallet finger injury using Tubiana’s classification system.

Each prompt was prefaced with, “I am a plastic and reconstructive surgeon who specializes in hand surgery. You are my colleague and I am discussing a case with you.” Each prompt included a fictionalized vignette and a specific hand injury diagnosis. Additionally, within each prompt was a request to classify the injury using a specific classification system and determine if this injury warrants surgical or nonsurgical (conservative) management. The deliberate inclusion of the specific diagnosis and classification system of interest was incorporated to focus the evaluation of the LLMs’ classification abilities, rather than their diagnostic skills, ensuring a uniform methodology and reducing the likelihood of LLMs using varying classification systems. Examples of prompts with LLM responses are depicted in Figure 1 and Figure 2. Each prompt was provided to ChatGPT-4 (OpenAI, San Francisco, CA, USA) and Gemini (Google, Mountain View, CA, USA) twice to ensure consistency and replicability, resulting in a total of 136 prompts. Additionally, each prompt was entered individually in a separate conversation to minimize the possibility of one answer affecting another. All prompts were provided on 10 March 2024, using the Google Chrome internet browser.

For the assessment of classification abilities, the LLMs were given points based on answer correctness. Completely correct classifications were awarded two points, partially correct classifications were awarded one point, and incorrect classifications were awarded zero points. Instances of partial correctness typically arose from either indecisiveness on the part of the LLM or from including subclassifications within the classification system. For example, in systems like the Gustilo–Anderson classification [20,33] for open fractures, which have subclassifications (e.g., Type IIIA, IIIB, and IIIC), partial correctness could result from selecting the wrong subclassification (e.g., selecting Type IIIB when the correct answer was Type IIIA) or from failing to specify the subclassification altogether (e.g., answering Type III when the correct answer was Type IIIA). Furthermore, if the LLM struggled to choose between two options, one of which was correct, it would still receive one point for partial correctness.

In our investigation into the LLM’s ability to differentiate between surgical and nonsurgical management options for hand injuries, we relied on clinically pertinent classification systems as our gold standard. These classification systems were chosen for their comprehensive treatment recommendations which were associated with each categorized injury. As such, the recommendations provided within the classification systems served as the definitive ‘correct’ answers for our study. Furthermore, to ensure the accuracy of our assessments, all responses were verified by a board-certified hand surgeon. By adhering to these established guidelines, we aimed to maintain consistency and objectivity in our evaluation process. This approach not only provided a clear framework for comparing LLM performance, but also ensured the clinical relevance and validity of our findings.

### 2.2. Data Collection and Analysis

Prompts and corresponding LLM responses were collected in Microsoft Excel (Redmond, WA, USA). Each response was graded based on classification correctness. These values were analyzed using descriptive statistics including the mean, standard deviation (SD), and range. Comparisons between ChatGPT and Gemini classification abilities were evaluated using paired, two-tailed *t*-tests. This test controls for variability across different clinical vignettes and allows for the detection of significant differences, without assuming directionality. Subgroup analyses were conducted to compare scores for each classification system. An alpha level of 0.05 was used to determine statistical significance.

In addition to classification accuracy, the evaluation of the LLMs’ capacity to recommend surgical versus nonsurgical management involved the calculation of sensitivity (also known as recall), specificity, positive predictive value (PPV; also known as precision), accuracy, and the F1 score. Sensitivity and specificity offer insights into the models’ ability to accurately identify true surgical and nonsurgical cases, respectively, while PPV and accuracy shed light on the precision and correctness of the models’ recommendations. Furthermore, the F1 score serves as a synthesized measure, capturing the balance between precision and recall, and offering a more nuanced understanding of the models’ overall performance. This multifaceted evaluation approach ensures that the strengths and weaknesses of the LLMs’ recommendations are thoroughly explored, providing valuable insights for healthcare providers and researchers alike in navigating the complexities of decision making in hand surgery.

## 3. Results

### 3.1. Classification Results

**Figure 3:**
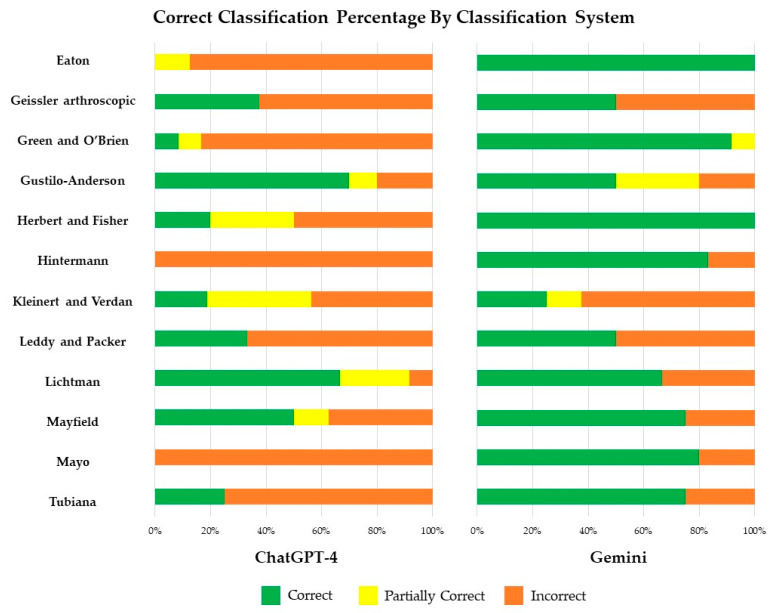
Percentage of correct classifications for each classification system for ChatGPT-4 (**left**) and Gemini (**right**).

In the classification of hand injuries, Gemini exhibited superior performance over ChatGPT, with an average score of 1.46 (SD 0.87), whereas ChatGPT yielded an average score of 0.67 (SD 0.87) (*p*-value < 0.001). Classification results are displayed in Table 1 and Figure 3. Gemini provided completely correct classifications for 96 (70.6%) hand injuries, but partially correct and incorrect for six (4.4%) and 34 (25.0%) injuries, respectively. ChatGPT provided correct, partially correct, and incorrect classifications for 36 (26.5%), 19 (14.0%), and 81 (59.6%) hand injuries, respectively.

ChatGPT exhibited its strongest performance in utilizing the Lichtman classification [38] for Kienböck disease (osteonecrosis of the lunate), correctly classifying eight cases (66.7%) with a mean score of 1.58 (SD 0.67). Additionally, its next best performance was in employing the Gustilo–Anderson classification [20,33] for open fractures, accurately categorizing seven cases (70%) with a mean score of 1.50 (SD 0.85). However, its performance was notably poorer when using Hintermann et al.’s classification [35] system for Gamekeeper’s thumb, where all of its classifications were incorrect. Similarly, ChatGPT struggled with the classification of scaphoid fractures, where it inaccurately classified all cases according to the Mayo classification [28,29] system. Furthermore, in classifying volar plate avulsion injuries using the Eaton classification [30] system, ChatGPT demonstrated unacceptable performance, failing to correctly classify any cases. In contrast, Gemini displayed superior classification capabilities, providing accurate classifications for all volar plate avulsion injuries using the Eaton classification [30] system and all scaphoid fractures using the Herbert and Fisher Classification [34]. Despite this, Gemini’s weakest performance was observed when classifying flexor tendon injuries using Kleinert and Verdan’s Zone classification [36] system. Notably, ChatGPT narrowly outperformed Gemini only in the Gustilo–Anderson classification [20,33] of open fractures, Kleinert and Verdan’s Zone classification [36] of flexor tendon injuries, and the Lichtman classification [38] for Kienböck disease (osteonecrosis of the lunate).

### 3.2. Surgical Management Results from Sensitivity Testing

Based on the clinical vignettes and classification, 98 (72.1%) injuries warranted surgical intervention, and 38 (27.9%) justified nonsurgical management. ChatGPT recommended surgical intervention for 108 (79.4%) cases, as compared to Gemini, which recommended surgery for only 89 (65.4%). The results of the sensitivity testing are shown in Table 2. ChatGPT demonstrated higher sensitivity (recall) in recommending surgical intervention when compared to Gemini (98.0% vs. 88.8%). However, Gemini demonstrated a specificity of 94.7%, which was higher than ChatGPT’s specificity of 68.4%. Additionally, Gemini demonstrated a PPV (precision) of 97.8%, which was higher than ChatGPT’s PPV of 88.9%. Both models exhibited comparable F1 scores, with ChatGPT achieving an F1 score of 0.932 and Gemini achieving an F1 score of 0.930.

### 3.3. Replicability Results

To ensure consistency, each of the 68 prompts was presented twice. Gemini’s classification response differed in six instances (8.9%). In contrast, ChatGPT showed more variability, with its classification changing in 17 cases (25.0%), indicating a lower level of consistency and replicability. Among ChatGPT’s changes, 12 (70.5%) resulted in a more accurate classification (e.g., changing from incorrect to partially correct, changing from incorrect to correct, or changing from partially correct to correct). In terms of recommending surgical or nonsurgical management, ChatGPT modified its answer for six (8.9%) injuries, five of which were corrected in the subsequent response. However, Gemini’s response changed only once (1.5%), albeit to the incorrect management choice.

## 4. Discussion

**Figure 4:**
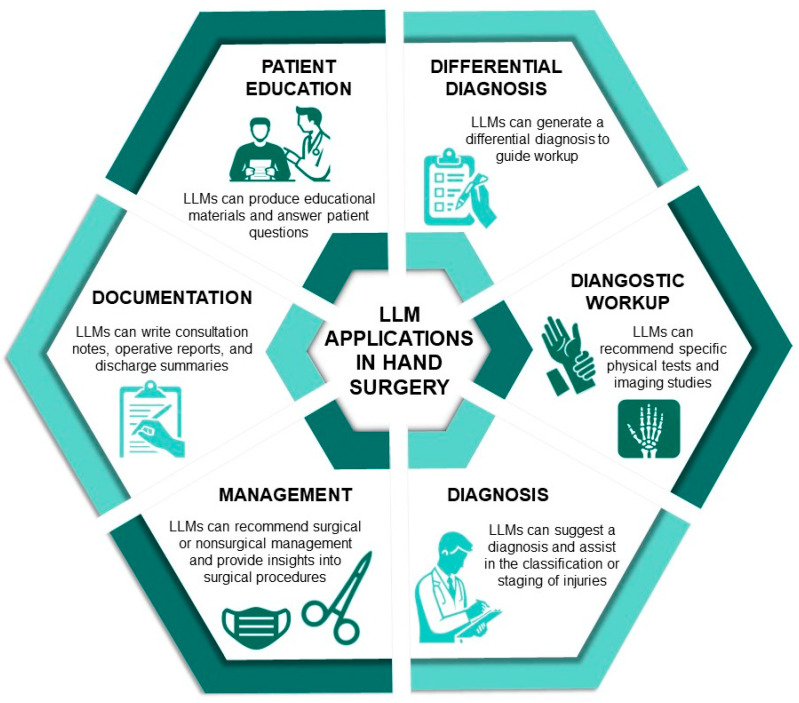
Applications of large language models (LLMs) in hand surgery.

With the correct classification of 70.6% of hand injuries, Gemini demonstrated superior performance to ChatGPT, which correctly classified just over a quarter of the injuries. ChatGPT’s poor performance, specifically when using the Eaton [30], Hintermann [35], and Mayo [28,29] classification systems, may suggest a lack of information relating to these classification systems in the dataset on which it was trained. The results of this study demonstrate Gemini’s superior performance over ChatGPT, which contrasts the few prior comparative studies involving Gemini, where ChatGPT was found to demonstrate greater accuracy [40,41]. Similarly, previous studies comparing ChatGPT to Gemini’s predecessor, Bard, have shown varied results, with some studies favoring ChatGPT [42,43,44,45] and others favoring Bard [46,47]. Without a definitive accuracy threshold, neither model is currently reliable enough as a classification tool to be used in clinical practice, but this situation is expected to change shortly. While neither ChatGPT nor Gemini were designed for medical use, the findings reveal promising potential in hand injury classification, which is just one of its potential applications in hand surgery (Figure 4). With the further expansion of datasets and the refinement of algorithms, these models are anticipated to reach the required level of accuracy for practical use.

The findings from sensitivity testing indicate that ChatGPT leans towards recommending surgical intervention more quickly, whereas Gemini takes a more cautious and conservative approach, showing hesitation in recommending surgical intervention. These differing behaviors carry significant implications, particularly in clinical decision-making scenarios. While ChatGPT’s promptness may offer a sense of urgency, it also raises concerns regarding the potential for the over-recommendation of surgical procedures. On the other hand, Gemini’s reluctance may contribute to a more conservative approach, minimizing the risk of unnecessary interventions, but possibly at the expense of timely action when surgical intervention is indeed warranted. Healthcare providers must carefully consider these nuances when incorporating AI decision-support tools into clinical practice, balancing the need for prompt action with the importance of exercising prudence and minimizing unnecessary interventions.

When compared to ChatGPT, Gemini also demonstrated greater consistency in its answers. This is not unsurprising, as previous studies [48,49,50] have reported concerns regarding ChatGPT’s consistency. To be a reliable medical resource, an LLM must ensure that its prompts and recommendations are reproducible, replicable, and consistent across diverse interactions and contexts. Achieving this ensures that healthcare providers can trust the model’s outputs consistently, thereby streamlining their decision-making processes [50]. This enhanced reliability not only instills confidence in the LLM’s capabilities, but also translates into more efficient and effective patient care, as clinicians can rely on the model to provide accurate and consistent guidance in various medical scenarios.

Although not the main focus of this study, it was evident that ChatGPT tended to generate lengthy and verbose responses, whereas Gemini provided more concise ones. In a clinical environment, where healthcare providers are frequently pressed for time and efficiency is paramount, the ability to deliver concise responses can be highly beneficial. Given that every minute counts in such settings, the succinctness of Gemini’s responses may offer a practical advantage, enabling healthcare professionals to quickly grasp essential information without unnecessary verbosity. In the high-pressure setting of the emergency department (ED), where healthcare providers are often inundated with urgent cases, the rapid delivery of concise, accurate information would be especially beneficial. Gemini’s capability to provide succinct responses can support emergency providers in quickly assessing and prioritizing cases, thus enabling efficient resource allocation and expediting critical interventions.

ED applications of LLMs thus far have mostly focused on evaluating ChatGPT’s ability to triage and diagnose. Berg et al. [48] examined ChatGPT’s capacity to generate differential diagnoses, concluding that, while it can aid clinicians, its inconsistent responses limit its potential to replace clinical judgment. Meanwhile, Fraser et al. [51] compared ChatGPT-3.5 and ChatGPT-4 in triage and diagnosis, finding that ChatGPT-3.5 possessed high diagnostic accuracy but insufficient triage abilities. ChatGPT-4 showed improved triaging capability but lower diagnostic accuracy. The authors advised against unsupervised patient use and advocated for efforts to improve diagnostic and triage accuracy. Further studies highlighting ChatGPT’s potential in emergency medicine include its role in suggesting diagnostic imaging [52] and providing diagnostic recommendations based on electrocardiography data [53]. Given that hand injuries are often first seen and evaluated in the ED [1,23,54], equipping emergency providers with resources like LLMs can help expedite triage and diagnostic workup.

The potential of LLMs to expedite diagnostic workup and management presents a promising solution for supporting emergency and primary care providers in managing hand injuries rapidly while waiting for a hand surgeon. This application could be especially advantageous in rural or underserved areas lacking on-site hand specialists. LLMs can empower these frontline providers to initiate diagnostic processes and treatment strategies, serving as a valuable resource until a hand specialist can evaluate the patient. This concept of using LLMs as a specialty consult has been previously discussed in the literature [50,51,55,56]. However, it is important to note that, while LLMs can support providers and bridge the gap between the initial presentation and hand surgery evaluation, they should not be used to replace an actual consultation with a hand specialist [1,11,13,24].

### 4.1. Ethical Considerations

As the integration of LLMs into medical practice becomes increasingly prevalent, it is imperative to address the ethical considerations and limitations associated with their use. While LLMs offer immense potential to enhance patient care and medical decision making in hand surgery, they also pose ethical challenges that necessitate careful attention. Upholding ethical principles, such as autonomy, beneficence, nonmaleficence, and justice, is paramount in the development and deployment of these models.

- Autonomy: Healthcare providers must ensure that LLMs respect a patient’s autonomy by facilitating informed decision making and respecting their preferences and values, especially throughout the surgical process [57,58]. A patient’s autonomy may be compromised if they are not adequately informed about the limitations, biases, and role of LLMs in their care [1,59].
- Beneficence: LLMs have the potential to significantly benefit patient care by providing timely and accurate information that can empower both healthcare professionals and patients to make more informed decisions. However, the implementation of LLMs must be guided by a commitment to maximizing these potential benefits while minimizing harm [48,59].
- Nonmaleficence: While LLMs can offer valuable assistance, they also carry inherent risks, including the potential for errors, biases, and misinformation [1,3,11,48]. Healthcare providers must critically evaluate and verify LLM-generated recommendations. Additionally, measures should be in place to mitigate the risk of LLMs propagating misinformation or perpetuating bias and healthcare disparities [57,59]. This entails the ongoing monitoring and evaluation of LLM performance, as well as efforts to address any identified issues or limitations.
- Justice: The fair distribution of resources requires equitable access to this technology and its benefits [2]. Failing to address LLM bias and disparities in LLM utilization could exacerbate existing inequities in healthcare access and outcomes [1,2,52,57]. Therefore, it is imperative for healthcare systems to implement policies and initiatives aimed at promoting equitable access to LLM technology.

By paying careful attention to issues such as data privacy, transparency in decision-making processes, liability, and the mitigation of biases, healthcare can navigate the integration of LLMs in a manner that prioritizes ethical integrity. By proactively addressing these ethical considerations, healthcare can harness the full potential of LLMs while safeguarding patient autonomy, well-being, and justice in medical practice.

### 4.2. Limitations

We acknowledge multiple limitations to the study and the generalizability of its results. The clinical scenarios depicted in this study adhered closely to textbook examples. This prompts the question: how would these models fare when faced with less straightforward vignettes? Real-life patient encounters frequently involve atypical or complex presentations, which may diverge from the expected norms. Models trained solely on textbook-like cases may struggle to accurately interpret and respond to the complexities inherent in real-world medical practice. Evaluating these models’ adaptability to a wide range of clinical scenarios is essential for their effectiveness. Failing to do so risks undermining their reliability and applicability in real-world healthcare settings, potentially compromising patient care outcomes. Therefore, future research endeavors should prioritize testing these models against a wider range of clinical vignettes to comprehensively assess their real-world utility and identify areas for improvement.

Although this study included 68 unique patient vignettes covering 12 classification systems, it is by no means comprehensive. While these vignettes offer valuable insights into the classification abilities of the models under examination, they may not fully encapsulate the diverse spectrum of hand injuries encountered in clinical practice. As such, the study’s findings should be interpreted within the context of its inherent constraints. Despite these challenges, our study serves as a starting point for future investigations to delve into these nuances and to advance our understanding of LLM performance in real-world clinical practice.

Furthermore, the success of using LLMs in medicine depends entirely on their ability to provide accurate and reliable information. The speed at which an LLM can respond to queries becomes irrelevant if it offers incorrect and potentially harmful recommendations. Misinformation can lead to adverse patient outcomes and the erosion of trust in technology and healthcare providers [9,57,60]. As previously mentioned, we acknowledge that neither ChatGPT nor Gemini was designed specifically with medical applications in mind. The datasets on which these models were trained likely lack significant medical information and data. This inherent limitation underscores the importance of further refining LLMs specifically for healthcare applications in order to mitigate such shortcomings.

### 4.3. Future Research and Next Steps

Moving forward, it is imperative to address the limitations highlighted in this study to maximize the potential of LLMs in healthcare. While ChatGPT and Gemini have exhibited promising capabilities in classifying hand injuries and offering management recommendations, there remains a need for further research to refine and enhance their performance for real-world clinical applications. One crucial area of focus for future investigation involves expanding the training datasets of these LLMs with more comprehensive medical information and clinical data. This would allow these models to better navigate the intricacies of medical decision making. Additionally, future studies should aim to evaluate the performance of ChatGPT, Gemini, and other LLMs in classifying atypical or complex hand injuries, as well as extending their assessment to encompass a broader spectrum of injuries and medical conditions. Most current studies focus exclusively on ChatGPT, but with Gemini’s superior performance in hand injury classification, further studies examining this model’s ability are warranted. By systematically examining LLM performance in diverse clinical scenarios, researchers can identify areas for improvement and tailor these models to address the specific challenges encountered in medical practice.

Furthermore, the advancement of LLMs in medicine necessitates ongoing research and development. By continually refining and validating AI tools like ChatGPT and Gemini, healthcare professionals can harness their full potential as invaluable resources in clinical practice. Further investigation into the use and performance of additional AI tools like RAG, especially when used in conjunction with LLMs, is indicated. Through collaborative interdisciplinary efforts between developers, healthcare institutions, and medical professionals, the development of robust and reliable medical-focused LLMs can pave the way for a new era of personalized and efficient healthcare delivery.

## 5. Conclusions

This study evaluates the performance of ChatGPT and Gemini in classifying hand injuries and suggesting management. While both models show potential, Gemini generally performs better than ChatGPT in classification, although not currently at a suitable level for current use. For treatment recommendations, ChatGPT leans towards recommending surgical intervention more readily, albeit with lower specificity than Gemini. These findings stress the need to carefully weigh the strengths and limitations of different LLMs when incorporating them into clinical practice. Both ChatGPT and Gemini hold promise as valuable resources for hand surgeons. This potential is expected to translate into enhanced diagnostic accuracy and treatment decisions, ultimately improving patient outcomes. However, further development and research are necessary to ensure the reliability of these models.

## Figures and Tables

**Table 1:** Hand injury classification results from the LLMs.

Classification System	LLM	Correct	Partially Correct	Incorrect	Mean Score	Standard Deviation
Eaton classification for volar plate avulsion injuries	ChatGPT-4	0	1	7	0.13	0.35
	Gemini	8	0	0	2.00	0.00
Geissler arthroscopic classification for carpal instability	ChatGPT-4	3	0	5	0.75	1.04
	Gemini	4	0	4	1.00	1.07
Green and O’Brien’s classification of thumb metacarpal fractures	ChatGPT-4	1	1	10	0.25	0.62
	Gemini	11	1	0	1.92	0.29
Gustilo-Anderson classification of open fractures	ChatGPT-4	7	1	2	1.50	0.85
	Gemini	5	3	2	1.30	0.82
Herbert and Fisher Classification of scaphoid fractures	ChatGPT-4	4	6	10	0.70	0.80
	Gemini	20	0	0	2.00	0.00
Hintermann et al.’s classification of ulnar collateral ligament (UCL) injury of the thumb	ChatGPT-4	0	0	12	0.00	0.00
	Gemini	10	0	2	1.67	0.78
Kleinert and Verdan’s Zone classification of flexor tendon injuries	ChatGPT-4	3	6	7	0.75	0.77
	Gemini	4	2	10	0.63	0.89
Leddy and Packer classification of avulsion injury of the flexor digitorum profundus (FDP)	ChatGPT-4	4	0	8	0.67	0.98
	Gemini	6	0	6	1.00	1.04
Lichtman classification of Kienböck disease (osteonecrosis the lunate)	ChatGPT-4	8	3	1	1.58	0.67
	Gemini	8	0	4	1.33	0.98
Mayfield classification for carpal instability	ChatGPT-4	4	1	3	1.13	0.99
	Gemini	6	0	2	1.50	0.93
Mayo Classification of scaphoid fractures	ChatGPT-4	0	0	10	0.00	0.00
	Gemini	8	0	2	1.60	0.84
Tubiana classification for mallet finger	ChatGPT-4	2	0	6	0.50	0.93
	Gemini	6	0	2	1.50	0.93
Total	ChatGPT-4	36	19	81	0.67	0.87
	Gemini	96	6	34	1.46	0.87

**Table 2:** Results of sensitivity testing.

Value	ChatGPT	Gemini
Sensitivity	0.980	0.888
Specificity	0.684	0.947
Positive Predictive Value (PPV)	0.889	0.978
Negative Predictive Value (NPV)	0.929	0.766
Positive Likelihood Ratio (LR+)	3.102	16.867
Negative Likelihood Ratio (LR−)	0.030	0.118
Accuracy	0.897	0.904
F1 score	0.932	0.930

## Data Availability

The data that support the findings of this study are available from the corresponding author upon reasonable request.
